# Machine Learning and Criminal Justice: A Systematic Review of Advanced Methodology for Recidivism Risk Prediction

**DOI:** 10.3390/ijerph191710594

**Published:** 2022-08-25

**Authors:** Guido Vittorio Travaini, Federico Pacchioni, Silvia Bellumore, Marta Bosia, Francesco De Micco

**Affiliations:** 1 School of Medicine, Vita-Salute San Raffaele University, 20132 Milan, Italy; 2 Department of Clinical Neurosciences, IRCCS San Raffaele Scientific Institute, 20132 Milan, Italy; 3 Bioethics and Humanities Research Unit, Campus Bio-Medico University of Rome, 00128 Rome, Italy; 4 Department of Clinical Affairs, Campus Bio-Medico University Hospital Foundation, 00128 Rome, Italy

**Keywords:** machine learning, recidivism, crime prediction, artificial intelligence, explainable artificial intelligence

## Abstract

Recent evolution in the field of data science has revealed the potential utility of machine learning (ML) applied to criminal justice. Hence, the literature focused on finding better techniques to predict criminal recidivism risk is rapidly flourishing. However, it is difficult to make a state of the art for the application of ML in recidivism prediction. In this systematic review, out of 79 studies from Scopus and PubMed online databases we selected, 12 studies that guarantee the replicability of the models across different datasets and their applicability to recidivism prediction. The different datasets and ML techniques used in each of the 12 studies have been compared using the two selected metrics. This study shows how each method applied achieves good performance, with an average score of 0.81 for ACC and 0.74 for AUC. This systematic review highlights key points that could allow criminal justice professionals to routinely exploit predictions of recidivism risk based on ML techniques. These include the presence of performance metrics, the use of transparent algorithms or explainable artificial intelligence (XAI) techniques, as well as the high quality of input data.

## 1. Introduction

Recidivism rates have a major impact on public safety and increase the cost of incarceration. An estimate of economic and social costs of recidivism has been provided by one study conducted in England and Wales on a 12-month follow-up, indicating GBP 18.1 billion for the 2016 criminal cohort [1]. This incidence is also influenced by the high rate of criminal recidivism, stated as high as 50% in many international jurisdictions [2]. In addition to the social costs, it is also important to mention the devastating consequences that recidivism causes for victims, communities and offenders as well as their own families, which are usually not even mentioned among the injured parties. So, it is very important to try to reduce the high rate of criminal recidivism and reduce these effects. 

A discipline that in recent years has provided an important contribution by attempting to provide for criminal recidivism is data science. Indeed, data science is the application of quantitative and qualitative methods to solve relevant problems and predict outcomes [3,4,5]. In particular, using the potential of risk assessment tools and machine learning (ML) algorithms in predicting the risk of criminal recidivism in order to reduce its spread has been outlined in literature since the 1920s [6]. Over the years, the methodologies improved becoming more reliable due to the development of various datasets and ML models able to support judicial decisions on probation, length of sentence or application of better rehabilitation strategies. ML models can indeed be applied in various criminal justice areas. At the group level, they can be profitably used to monitor or predict the effects of criminal justice policies on reducing recidivism. At the individual level, they can be applied to support judicial decisions, assessing specific risk of recidivism. 

This review aims to deepen these debated topics. Indeed, despite the extensive development of these approaches and a promising assessment of the reliability of ML models to support criminal justice [7], they are still underutilized in judicial practice [8]. This stems from the controversial opinions debated in the scientific and jurisprudential literature [9,10,11,12]. The foremost reason is the fear of delegating decisions entirely to an algorithm, as reported by Rees “we would be entitled to feel uneasy, even if presented with compelling evidence that, on average, the machines make better decisions than the humans” [13]. Despite the perplexities, it is hard to imagine our future ignoring the potentials of ML model’s application [14]. Indeed, the ability of ML systems to predict complex outputs with high certainty has led them to become increasingly pervasive and has received growing attention in recent years to optimize public policy and criminal justice [15,16,17,18]. Thus, researchers have sought to improve prediction scales by looking for strong indicators that predict different types of recidivism thus improving the accuracy of the ML model. However, it is also important to focus on previously overlooked aspects and interactions, such as standardizing data collection and applying data pre-processing, two process that can increase the performances of ML models. The different variables considered for the prediction of recidivism and the constantly evolving ML techniques have raised the need for clarity in the vast literature that has rapidly followed over the years. Leveraging the potential of a review written according to the Preferred Reporting Items for Systematic Reviews and Meta-Analyses (PRISMA) Statement [19], this study aims for the first time to delineate the state of the art in recidivism prediction techniques with a critical analysis of their potentialities and limitations.

## 2. Materials and Methods

### 2.1. Eligibility Criteria

The methods for systematic review have been structured according to the PRISMA Statement. No language, publication date, or publication status restrictions were imposed. The features of included studies are the following: (1) the aim of the study is to predict recidivism; (2) the study has explicit accounting of data collection methods; and (3) the study has proper description of the methodologies, including the applied machine learning methods.

In contrast, the authors did not include studies in which one or more of the following criteria were satisfied: (1) the main purpose of the study is to reduce the bias of the ML model (e.g., race-based bias); (2) the study has the aim to predict the psychiatric characteristics in the reoffender (e.g., mental illness); and (3) the study lacks the accuracy (ACC) or the Area Under the Curve (AUC) metrics necessary to evaluate machine learning models.

### 2.2. Information Sources and Search Strategy

Studies have been selected from two online databases: Scopus and Pubmed. We used the following equation searching by title, abstract and keywords for: ((“crim*”) OR (“offen*”) OR (“violat*”)) AND ((“recidiv*”) OR (“relapse”)) AND ((“machine learning”) OR (“artificial intelligence”) OR (“deep learning”)) resulting in a total of 79 bibliographic records. References listed in the included papers have also been examined to identify studies meeting our inclusion and exclusion criteria. The search was last performed in January 2022. We first screened titles and excluded those clearly not meeting the eligibility criteria. Then abstracts were examined, and lastly, full texts were read, eventually leading to the inclusion or exclusion of the papers according to the above described criteria. The screening of the literature was performed in blind by two investigators (S.B. and F.P.) In the case of disagreement, a third reviewer (M.B.) assessed the paper to achieve a consensus.

### 2.3. Assessment of Risk of Bias

The ROBIS tool was used to assess the risk of bias of the included systematic reviews [20]. The ROBIS tool is a method for assessing bias in systematic reviews that consists of three phases: (1) assessing relevance (optional); (2) identifying concerns with the review process; and (3) judging risk of bias in the review. Phase two involves assessing the review across four domains: (1) study eligibility criteria; (2) identification and selection of studies; (3) data collection and study appraisal; and (4) synthesis and findings. The third phase summarizes the concerns identified during phase two. The first two phases were performed independently by two authors (SB, FP) and resulting discrepancies were addressed by a third author (GT or MB).

## 3. Results

### 3.1. Study Selection

A total of 16 duplicates were identified and removed from the 79 preliminary results. In addition, 33 articles were removed by looking at the title and abstract only since they did not match the criteria. The remaining papers were selected or excluded after a comprehensive analysis of the full text. Among those papers, 18 were excluded since they did not satisfy eligibility criteria for the following reasons:

- The purpose of fourteen papers was to reduce model bias;
- One paper assessed only the psychiatric characteristic of repeat offenders;
- Three papers did not clearly describe the methodology.

**Figure 1:**
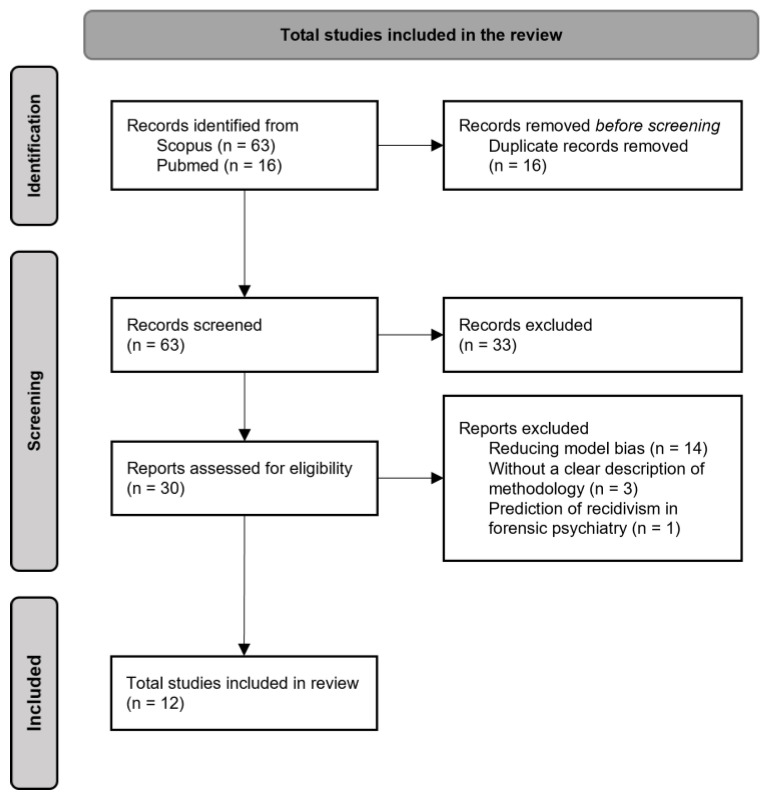
Flowchart showing the process of inclusion of publications.

A total of 12 studies were finally selected. Figure 1 summarizes the results of the study selection, represented as a PRISMA flow diagram obtained following the guidelines published by Page and colleagues [19]. 

### 3.2. Study Characteristics

To present the results of the studies, we divided them into three sections. The first one analyses dataset and ML techniques applied within the study. We first focused on the characteristics of datasets. Then we checked whether in the studies the authors have used ML techniques such as data pre-processing or cross validation (CV). The first one is a technique to transforming raw data into an understandable format for the ML models. The CV, instead, is a technique to estimate if the ML models are able to correctly predict data not yet observed. This first section is important because different datasets and different ML techniques can greatly influence the final performance of ML models. In the second section, we analysed the type of recidivism that each study aimed to predict. Then we selected the ML model that in the study obtained the best performance. Finally, in the third section, by dividing the studies into four categories based on their aim, we compared the performance of each ML model based on specific metrics.

### 3.3. Characteristics of Dataset and ML Techniques

The main features of the considered studies are listed in Table 1.

The datasets are different in each study. Two of them used only data from the correctional institution or the justice system. The first one was based on 3061 youth charged with a sexual offense in Florida who have been monitored over two years after the initial charge to determine sexual recidivism [26]. The aim of Ozkan and colleagues was to examine whether ML models could provide a better prediction than classical statistical tools. Thus, the authors took advantage of the statistical models using several predictor variables, including historical risk assessment data and a rich set of developmental factors for all youth reported for delinquency by the Florida Department of Juvenile Justice (FDJJ). The second study published by Butsara [21] used a dataset from a central correctional institution for drug addicts and a central women’s correctional institution in Thailand. The sample consists in 300 male and 298 female inmates. The authors proposed a method to find the crucial factors for predicting recidivism in drug distribution and investigated the power of ML in predicting recidivism. 

Five papers refer to risk assessment tools created specifically to predict recidivism across the country. Among them Karimi-Haghighi and Castillo used RisCanvi, a risk assessment protocol for violence prevention introduced in the Catalan prison during 2009 in which professionals conducted interviews resulting in the creation of a risk score through some risk elements [25]. The elements are included in five risk areas of prisoners: criminal/penitentiary, family/social, clinical and attitudinal/personal factors. The dataset used includes 2634 cases. The Duwe and Kim study considers 27,772 offenders released from Minnesota prisons between 2003 and 2006 [22]. The authors used a dataset from the Minnesota Screening Tool Assessing Recidivism Risk (MnSTARR), which assesses the risk of five different types of recidivism, taking advantage of the Minnesota Sex Offender Screening Tool-3 (MnSOST-3), used to analyse sexual recidivism risk for Minnesota sex offenders. Tollenaar and colleagues in two different studies used the StatRec scale with static information from the Dutch Offender Index (DOI) [31,32]. In both studies, the recidivism prediction is divided into three categories: general, criminal and violent recidivism. The dataset is based on offenders over the age of 12 found guilty during a criminal case that ended in 2005. In the more recent one, the authors also included public access data from the North Carolina prison in the dataset to investigate the generalizability of the results. These data feature all individuals released from July 1977 to June 1978 and from July 1979 to June 1980. Both cohorts were tested with ML models, but for this review we consider only the 1977–1978 data excluding the Tollenaar’s 2019 study [32] because the 1980 cohort showed a worse calibration probability. The dynamic elements of the Finnish Risk and Needs Assessment Form (RITA) were used by Salo and colleagues [27] to predict general and violent recidivism. The sample included 746 men sentenced to a new term of imprisonment. All individuals must have the full RITA, which considers 52 items such as aggression, alcohol problems, drug use, work problems, coping with economic problems and resistance to change. 

Another study that combined statistical features with other specific risk assessment tools is by Tolan and collaborators [30]. The authors compared different combinations of datasets and ML models in terms of AUC and then showed the results with Structured Assessment of Violence Risk in Youth (SAVRY) features in which the ML model outperforms. The SAVRY is a violent risk assessment tool including 24 risk factors and 6 predictive factors. These risk factors are divided into historical, individual and social/contextual categories. The data analysed in the study were extracted from the Catalonia juvenile justice system and included 853 juvenile offenders between the ages of 12 and 17 who finished a sentence in 2010 and were subjected to a SAVRY analysis. 

Only one study used the Historical, Clinical and Risk Management–20 (HCR-20) with 16 other clinical and non-clinical risk assessment factors in order to determine the likelihood of recidivism among first-time offenders (FTOs) [28]. Data were collected from various prisons in the Indian state of Jharkhand. The study was conducted on 204 male inmates aged between 18 and 30, most of them below the poverty line. 

Finally, it is important to mention the paper of Haarsma and his working group [24] in which the authors used the NeuroCognitive Risk Assessment (NCRA), a neurocognitive test-based risk assessment software able to measure key criminogenic factors related to recidivism. In this study, 730 participants in the Harris County Department of Community Supervision and Corrections self-administered the NCRA. The individual’s recidivism risk score by NCRA combined with a set of demographic features was quantified using a ML model. 

After compiling the dataset, each study used different ML techniques to analyse and improve the reading of the data and results. Four studies used pre-processing to improve the datasets. Two of them preferred a feature selection technique [24,26]. One study used a generic data standardization and then applied feature selection [21]. The last one used ANOVA to identify relevant attributes for the current dataset [28]. 

Another relevant aspect to mention is the author’s choice to use a ML technique called cross validation (CV) in order to estimate the ability of ML models to generalize to data not yet observed. Among the studies included in the review, nine used CV.

### 3.4. Aim of the Studies and ML Model Applied

For a better comparison of the studies, it is possible to perform a sorting by the type of recurrence they aim to predict. The sorting leads to four categories: general [22,23,24,28,31,32], sexual [31,32], violent [22,25,31] and all other recidivism. The last category includes studies that considered a specific type of crime [21] or referred only to males [27] or youth [26,29,30].

The previously observed datasets (Table 1) were used to train ML models to generate recidivism predictions. There are different types of models that you can select based on the available data and type of target you want to predict. So, all the studies compared different models to obtain the best results in predicting recidivism. The metrics used to compare the models were accuracy (ACC) and area under curve (AUC). The ACC is used to measure how often the algorithm correctly classifies a data point and represents the ratio of correctly classified observation to the total number of the predictions. The AUC measures the ability of the ML models to distinguish recidivism from non-recidivism. Both metrics provide a result in the range [0,1]. When the score is near 0, it means that ML models have the worst ability to predict, a score close to 0.5 means that the models randomly predict the probability of recidivism. On the other hand, a score close to 1 means that ML models have a perfect ability to distinguish recidivism from non-recidivism. 

In this review, for each study, we considered only the ML models that performed better, according to the author’s observation (Table 2). The most used ML model is the logistic regression [21,30,31,32] and two of its variants, the LogitBoost [22] and the generalized linear models with ridge and lasso regularization (Glmnet) [24]. The second most popular model is the random forest [23,26,27,33]. The other ML models to mention are multi-layer perceptron (MLP) [25], linear discriminant analysis (LDA) [31] and penalized LDA [32].

### 3.5. Results of Syntheses

Table 3, Table 4, Table 5 and Table 6 describe the results for the analysis of ACC and AUC in general, sexual, violent and all the other recidivism in which all ML models perform well. The ACC scores are in the range [0.65, 0.96], and the AUC scores are in the range [0.69, 0.78]. The highest scores have been highlighted in Table 3, Table 4, Table 5 and Table 6. Table 3 shows the results obtained from the different studies predicting generic recidivism. The ensemble model trained with an HCR-20+ dataset leads to improved performance and seems to be the most effective method in terms of the ACC [28]. Considering the AUC, the logistic regression yields the highest score with the use of the MnSTARR+ and StatRec datasets [22,31].

In Table 3, Table 4 and Table 5, the comparison between the StatRec and DOI datasets shows that they produce the same results except for general recidivism with a difference of 0.05 [31,32]. However, this result was not surprising since both studies used data from the Dutch Offender’s Index.

**Table 4:** Evaluation sexual recidivism.

	StatRec	DOI
ACC	**0.96**	**0.96**
AUC	0.73	**0.77**

AUC: area under curve; ACC: accuracy; StatRec: static recidivism risk (Static Recidiverisico); DOI: Dutch Offender’s Index. The highest scores are highlighted in bold.

In contrast, for violent recidivism (Table 5), the multi-layer perceptron (MLP) trained with the RisCanvi dataset performed better than the other techniques [26].

**Table 5:** Evaluation violent recidivism.

	RisCanvi	StatRec	DOI
ACC		**0.78**	**0.78**
AUC	**0.78**	0.74	0.74

AUC: area under curve; ACC: accuracy; RisCanvi: risk assessment protocol for violence prevention introduced in the Catalan prison; StatRec: static recidivism risk (Static Recidiverisico); DOI: Dutch Offender’s Index. The highest scores are highlighted in bold.

Table 6 shows the results of studies that include all other recidivism. These studies are difficult to compare because each one has a different sample type, such as juvenile offenders or males or aims to predict a specific recidivism (e.g., recidivism in drug distribution). However, we observed that the results obtained reflect an overall effectiveness of prediction models. The Thailand dataset has a particular relevance with an ACC of 0.90 obtained with logistic regression [21]. In term of AUC, a model obtained a significant score of 0.78 using the RITA+ dataset trained with random forest [27]. 

**Table 6:** Evaluation all the other recidivism.

	Thailand	FDJJ	RITA+	YLS/CMI	SAVRY+
ACC	**0.90**			0.65	
AUC		0.71	**0.78**	0.69	0.71

AUC: area under curve; ACC: accuracy; Thailand: Thailand: data by central correctional institution for drug addicts and central women correctional institution in Thailand; FDJJ: Florida Department of Juvenile Justice; RITA+: Finnish Risk and Needs Assessment Form *+* static predictors; YLS/CMI: Youth Level of Service/Case Management Inventory 2.0; SAVRY+: Structured Assessment of Violence Risk in Youth + static features. The highest scores are highlighted in bold.

### 3.6. Factors Involved in Predicting Recidivism

In some of the studies considered in this review, the authors described the variables that contributed most to the final evaluation. Some considerations about these results are discussed in Section 4. Below we report the variables most implicated in the results of each model (when provided by the authors).

Four top factors are identified: royal pardons or suspension, first offending age, encouragement of family members and frequency of substance abuse [21]. Among the items in the LS/CMI they identify: Items A18 (charge laid, probation breached, parole suspended during prior community supervision), A 14 (three or more present offenses), A 423 (could make a better use of time) and A 735 (current drug problem) [23]. A total of 13 tests are selected within NCRA, in particular: balloon analog risk task (time collected), point-subtraction aggression paradigm (grow, punish ratio), reading the mind through the eyes (correct, time median), emotional stroop (test time, black time, icon color time, Pos Neg time) and Tower of London (aborted, dup moves, illegal moves first move frac) [24]. The stronger predictors in this model (sexual recidivism) are prior felony sex offense referrals, number of prior misdemeanor sexual misconduct referrals and number of prior felony offenses [26]. Among the dynamic factors extracted from RITA’s items, the most important seem to be problem’s managing one’s economy for general recidivism and aggressiveness for violent recidivism [27]. The most significant variables affecting the model accuracy is the total YLS score followed by difficulty in controlling behavior, age at first arrest, history of running away and family circumstances [29]. The most important features for the logistic regression model include almost all static features (sex, ethnicity, age at main crime, crime in 10 years, etc.,) and only one SAVRY feature (evaluation of the expert). For the MLP model, all static features are more important than SAVRY features [30]. For general recidivism (logistic regression model), the most powerful predictors are age, conviction density, specific offense types (property offence and public order offence), the number of previous offences and home country. Considering violent recidivism (logistic regression model), the largest effects can be seen in the number of previous convictions, the most severe offence type (property crime with violence), offence type present in the index case (property crime without violence, public order, other offence) and country of origin. Lastly, for sexual recidivism (linear discriminant analysis), three main coefficients are identified: previous sexual offences and country of origin have the greater positive affect on the probability of sexual recidivism, while the number of previous public prosecutor’s disposals has the largest negative effect [31].

### 3.7. Reporting Biases

In this systematic review, the datasets and ML models included have been compared simultaneously. However, for a critical evaluation of the results of ML models, it is also necessary to focus on the type of dataset used to train the model. 

In each study, the method of data collection is different. In some papers, the data come from the countries’ institutions, while in others they come from the checklists of risk assessment tools or neurocognitive tests. Consequently, datasets have different characteristics such as sample size, mean age, type of crime and years of recidivism follow-up. These variables can significantly modify the assessment of the ML model [34].

**Figure 2:**
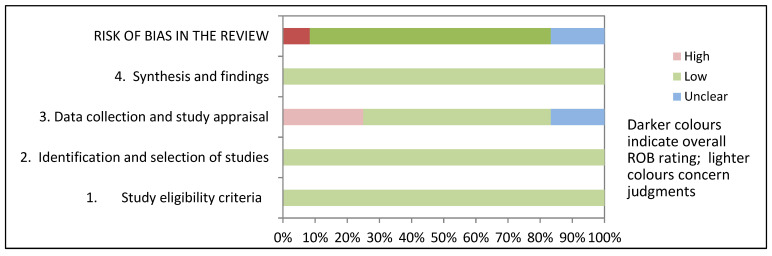
Graphical display for ROBIS results.

Another relevant aspect to mention is that not all the papers declare the use of data pre-processing or are clearly explicit about the process used. As mentioned above, we applied the ROBIS assessment to highlight any possible systematic distortion. Table 7 and Figure 2 summarize the risk of bias. In detail, in Table 7, for each study the relative risk of bias in each of the four domains is assigned on a three-level scale indicating “low” or “high” risk of bias or “unclear” risk when no information is available to judge a particular item. Figure 2 depicts the relative risk of bias (as in table N, from “high” to “low” and “unclear” risk) among all the included studies for each domain assessed as well as the “overall risk”. Of the considered studies, nine were evaluated as low risk overall, one as high risk, and two studies resulted in an unclear risk. The main concerns relate to the inhomogeneity of the samples and the poor description of data pre-processing and analysis which makes it harder to compare different ML techniques.

## 4. Discussion

The current state of data science reveals that ML algorithms may be very efficient. Accordingly, the results of the papers considered in this review show that each ML model has a good performance. Taking into consideration the ACC, the mid-score is 0.81. In contrast, considering the AUC the mid-score is 0.74. Considering that the range of ACC and the AUC are from 0 (no predictability) to 1 (perfect predictability), both mid-scores show a good predictability of the models. Some studies use a risk assessment for a specific type of recidivism, crime or population, while others measure the risk of general recidivism. However, comparing the two types of studies, no significant differences emerge. The only difference observed in this review is an increase in ACC of 0.03 for the specific type of recidivism. Thus, there is no evidence that the use of a more specific risk assessment could significantly improve the ability to predict criminal recidivism. 

This review compares different ML methods, models and datasets used to predict recidivism. Analysing the available models, the most common technique used in these papers to predict recidivism is logistic regression, while more complex algorithms are less common. In terms of performance, a simpler prediction model such as logistic regression and more complex ones such as the random forest show similar predictive validity and performance. What emerges from the results is that it does not seem essential to focus on model complexity to improve the predictability of criminal recidivism.

However, literature analysed in this systematic review pinpointed some limitations. First of all, the performance of each model does not depend only on the ML model or the dataset. The method of data collection and data pre-processing are also important. Both are aspects that can significantly affect model performance [34,35]. We observed that in the literature, it is uncommon to focus on data pre-processing techniques, which makes it difficult to compare different studies. In addition, all ML models used in the literature return a binary result, and for many of them, depending on the model and its description, it is difficult to understand which variables most influenced the final assessment. As a matter of fact, since the use of risk assessments has become more widespread and successful, this issue also emerged in recent studies in which the possibility of racial bias has been highlighted [36]. Age estimation-related biases, which in the forensic field are of significant relevance, should also be considered [37,38,39].

In Section 3.6, we reported the variables that have the greatest impact on the assessment of the risk of recidivism for each model. In this regard, it is good to make some considerations. As specified by the authors themselves, the most important variables for the final evaluation should be assessed limited only to the ML model used and to the specific dataset of that ML model. Hence, the results may not generalize to more specific subsamples or different subpopulations [31]. Moreover, for the final evaluation, it is not always possible to consider the single variables separately since the ML algorithms take into account the way in which the individual variables are combined [23]. Lastly, the complexity of the models that use interactions and nonlinear effects makes it challenging to explain individual-level predictions, and the difficulty grows along with the complexity of the model [22].

Using the ML method for risk assessment in criminal justice to predict recidivism has increased in recent years [14,23]. However, it is still a controversial topic due to the large amount of research on algorithmic fairness [40]. The purpose of this review is to analyse the state of the art of the techniques applied to predict criminal recidivism. Clearly, we did not aim to show the perfect prediction of the ML method nor to claim that it is possible to rely solely on the ML model to predict recidivism. Conversely, we highlight the strengths and limitations of data science applied to the humanities. 

First, we would like to point out that it would be important to pay more attention to the dataset and data processing. Taking a few steps back and focusing on these aspects could improve model performance and reduce possible bias [35]. 

Moreover, in order to facilitate comparison, it would be useful to learn to compare models by having the same evaluation metrics available. Considering metrics available in the analysed papers, we observed a good overall performance of the models. This allows us to emphasize the concrete support that these tools can bring to human judgment, which is also not free of bias [41]. The binary result is a limitation for this approach, as well as algorithmic unfairness [17]. 

The latest machine learning models are like ‘black boxes’ because they have such a complex design that users cannot understand how an AI system converts data into decisions [42]. The lack of accessibility of the models and algorithms used in judicial decisions could undermine the principles of transparency, impartiality and fairness and lead to the development of discrimination between individuals or groups of individuals [43]. It would be useful to develop transparent algorithms or use explainable AI. The explainable AI consists in AI systems that can explain their rationale to a human user and characterize their strengths and weaknesses [44]. With these techniques, we could know how much each variable affected the outcome, helping to form knowledgeable opinions usable from criminal justice professionals to motivate their decision [45,46]. In this regard, it would be useful to use a human-in-the-loop approach that leverages the strengths of collaboration between humans and machines to produce the best results, reinforcing the importance of the synergistic work [47,48]. 

## 5. Conclusions

The implementation of quantitative and qualitative methods to predict criminal recidivism could be a useful tool in the field of criminal justice [3,4,5] However, although research in this area is steadily increasing [7], its use in judicial practice is still limited [8] due to the controversial views [9,10,11,12]. This systematic review shows the state of the art regarding the application of ML techniques to the risk of reoffending and highlights key points useful for criminal justice professionals to exploit these new technologies. Each method applied achieves good performance, with an average score of 0.81 for ACC and 0.74 for AUC. However, the application of artificial intelligence in this field is still a controversial topic due to the significant critical issues [37]. To overcome these critical issues, it will be imperative to face and overcome a new challenge, that of making algorithms transparent and accessible so that the application of these new technologies contributes to decisions based on the principles of transparency, impartiality and fairness. In this regard, the integration of methods from the natural and social sciences according to a systemic orientation would allow the correlation between e-tech data and the human interpretation of the same [49], keeping the human operator at the head of the human–computer system in accordance with the integrated cognitive system [50].

The use of artificial intelligence in judicial proceedings and the resulting decision-making processes will be a field of wide reflection among scientists, jurists and bioethicists. This systematic review is a thorough synthesis of the best available evidence, but it is also a contribution in a field that presents many ethical, deontological and legal critical issues in the state of the art. 

## Figures and Tables

**Table 1:** Dataset combined and ML techniques applied.

Authors	Dataset Combined	ML Techniques
Butsara et al. (2019) [21]	Data by central correctional institution for drug addicts and central women correctional institution in Thailand	Data standardization + Feature selection and CV
Duwe and Kim (2017) [22]	Minnesota Screening Tool Assessing Recidivism Risk (MnSTARR) + Minnesota Sex Offender Screening Tool-3 (MnSOST-3)	CV
Ghasemi et al. (2021) [23]	Level of Service/Case Management Inventory (LS/CMI)	CV
Haarsma et al. (2020) [24]	NeuroCognitive Risk Assessment (NCRA) + demographic feature set	Feature selection + CV
Karimi-Haghighi and Castillo (2021) [25]	RisCanvi	CV
Ozkan et al. (2019) [26]	Florida Department of Juvenile Justice (FDJJ)	Feature selection
Salo et al. (2019) [27]	Finnish Risk and Needs Assessment Form (Riski-ja tarvearvio [RITA]) Finnish Prisoner Database + static predictors	CV
Singh and Mohapatra (2021) [28]	HCR-20 + clinical and non-clinical risk assessment factors	ANOVA + CV
Ting et al. (2018) [29]	Youth Level of Service/Case Management Inventory 2.0 (YLS/CMI)	
Tolan et al. (2019) [30]	Structured Assessment of Violence Risk in Youth (SAVRY) + static features	CV
Tollenaar et al. (2013) [31]	StatRec with Dutch Offender’s Index	
Tollenaar et al. (2019) [32]	Dutch Offender’s Index (DOI)	CV

CV: cross validation; ANOVA: analysis of variance.

**Table 2:** Purpose of datasets, ML models and their evaluation.

Dataset	Type of Recurrence	Purpose	ML Model	Evaluation Metrics	Evaluation Value
Thailand	Other	Recidivism in drug distribution	Logistic Regression	ACC	0.90
MnSTARR+	General	General recidivism	LogitBoost	ACC AUC	0.82 0.78
LS/CMI	General	General recidivism	Random Forest	ACC AUC	0.74 0.75
NCRA+	General	General recidivism	Glmnet	AUC	0.70
RisCanvi	Violent	Violent Recidivism	MLP	AUC	0.78
FDJJ	Sexual	Sexual recidivism in Youth	Random Forest	AUC	0.71
RITA+	Other	General and violent recidivism in male	Random Forest	AUC	0.78
HCR-20+	General	General recidivism	Ensemble model with NBC, kNN, MLP, PNN, SVM	ACC	0.87
YLS/CMI	Other	General recidivism in Youth	Random Forest	ACC AUC	0.65 0.69
SAVRY+	Other	Violent recidivism in youth	Logistic Regression	AUC	0.71
StatRec	General	General Recidivism	Logistic Regression	ACC AUC	0.73 0.78
	Sexual	Sexual recidivism	LDA	ACC AUC	0.96 0.73
	Violent	Violent recidivism	Logistic regression	ACC AUC	0.78 0.74
DOI	General	General recidivism	L1–Logistic Regression	ACC AUC	0.78 0.73
	Sexual	Sexual recidivism	L1–Logistic Regression	ACC AUC	0.96 0.77
	Violent	Violent recidivism	Penalized LDA	ACC AUC	0.78 0.74

ACC: accuracy; AUC: area under the curve; Thailand: data by central correctional institution for drug addicts and central women correctional institution in Thailand; MnSTARR+: Minnesota Screening Tool Assessing Recidivism Risk + Minnesota Sex Offender Screening Tool-3; LS/CMI: Level of Service/Case Management Inventory; NCRA+: NeuroCognitive Risk.

**Table 3:** Evaluation general recidivism.

	MnSTARR+	LS/CMI	NCRA+	HCR-20+	StatRec	DOI
ACC	0.82	0.74		**0.87**	0.74	0.78
AUC	**0.78**	0.75	0.70		**0.78**	0.73

AUC: area under curve; ACC: accuracy; MnSTARR+: Minnesota Screening Tool Assessing Recidivism Risk + Minnesota Sex Offender Screening Tool-3; LS/CMI: Level of Service/Case Management Inventory; NCRA+: NeuroCognitive Risk Assessment + demographic feature set; HCR-20+: Historical, Clinical and Risk Management–20 + clinical and non-clinical risk assessment factors; StatRec: static recidivism risk (Static Recidiverisico); DOI: Dutch Offender’s Index. The highest scores are highlighted in bold.

**Table 7:** Tabular presentation for ROBIS results.

	Phase 2				Phase 3
Review (Name, Year)	1. Study Eligibility Criteria	2. Identification and Selection of Studies	3. Data Collection and Study Appraisal	4. Synthesis and Findings	Risk of Bias in the Review
Butsara et al. (2019) [21]	☺	☺	☹	☺	☹
Duwe and Kim (2017) [22]	☺	☺	☺	☺	☺
Ghasemi et al. (2021) [23]	☺	☺	?	☺	☺
Haarsma et al. (2020) [24]	☺	☺	☺	☺	☺
Karimi-Haghighi and Castillo (2021) [25]	☺	☺	☺	☺	☺
Ozkan et al. (2019) [26]	☺	☺	☹	☺	?
Salo et al. (2019) [27]	☺	☺	?	☺	☺
Singh and Mohapatra (2021) [28]	☺	☺	☺	☺	☺
Ting et al. (2018) [29]	☺	☺	☺	☺	☺
Tolan et al. (2019) [30]	☺	☺	☹	☺	?
Tollenaar et al. (2013) [31]	☺	☺	☺	☺	☺
Tollenaar et al. (2019) [32]	☺	☺	☺	☺	☺

☺ = low risk; ☹ = high risk and ? = unclear risk.

## Data Availability

Not applicable.
